# Dislocation of the PreserFlo MicroShunt During a Postsurgical Needling Procedure

**DOI:** 10.7759/cureus.47356

**Published:** 2023-10-19

**Authors:** Kana Murakami, Mizuki Iida, Ayaka Shimada, Sho Ichioka, Akiko Harano, Aika Tsutsui, Masaki Tanito

**Affiliations:** 1 Department of Ophthalmology, Shimane University Faculty of Medicine, Izumo, JPN

**Keywords:** surgical complication, device dislocation, bleb needling, preserflo microshunt, glaucoma surgery

## Abstract

We report a case of PreserFlo MicroShunt (PFM) dislocation following a postsurgical needling procedure. A 58-year-old woman underwent PFM implantation for exfoliation glaucoma in her left eye (OS). There were no intraoperative complications. Preoperatively, her best-corrected visual acuity (BCVA) was 0.6, and her intraocular pressure (IOP) was 25 mmHg with three antiglaucoma medications in the OS. On postoperative day 21, the IOP was 21 mmHg OS, and the filtration bleb had shrunk. A needling procedure was performed using a sharp 26-gauge needle to lower the IOP. On postoperative day 29, the BCVA was 0.02, and the IOP was 60 mmHg OS. Gonioscopy revealed no device tip in the anterior chamber, and peripheral anterior synechia was observed at the site of PFM insertion. Anterior segment optical coherence tomography showed a dislocated device in the subconjunctival space. On postoperative day 35, the dislocated PFM was removed, and a new device was inserted. Following the reoperation, no further complications were observed, and bleb formation was obtained. In conclusion, like other glaucoma filtering surgeries, PFM may require postsurgical needling procedures. Needling procedures may cause PFM dislocation and IOP rise, resulting in the requirement for further IOP-reducing procedures.

## Introduction

The PreserFlo MicroShunt (PFM) (Santen Pharmaceutical, Co., Ltd., Osaka, Japan) is a glaucoma surgical device used for filtration surgery, aiming to reduce intraocular pressure (IOP) by draining aqueous humor from the anterior chamber (AC) into the subconjunctival space [1]. Compared to trabeculectomy, although the effectiveness in lowering IOP might be lower [2], filtration surgery with PFM has the merit of being an easier surgical procedure because there is no need to create a scleral flap or perform an iridectomy [1]. Compared to trabeculectomy, recent reports suggest a safer postoperative profile of PFM with a non-significant difference in IOP reduction [3,4].

When the size of the filtering bleb was reduced postsurgically following PFM implantation, it was treated with a needling procedure [5] or bleb revision [6], similar to trabeculectomy. Complications associated with needling procedures have been reported in eyes implanted with PFM, including blood reflux into the AC [7], conjunctival erosion [8], and endophthalmitis [9]. In this report, we present a case of PFM device dislocation complicated by a postsurgical needling procedure.

## Case presentation

A 58-year-old woman was undergoing ophthalmic treatment for exfoliation glaucoma. Her ophthalmologic history included treatment for idiopathic choroidal neovascularization and macular edema in the left eye (OS) with sub-Tenon steroid and intravitreal bevacizumab injections approximately 20 years earlier. Despite the use of antiglaucoma medications (latanoprost once/day and timolol and dorzolamide twice/day), her IOP was elevated, and glaucoma surgery was scheduled for the OS. The preoperative best-corrected visual acuity (BCVA) was 1.2 in the right eye (OD) and 0.6 OS, and IOPs were 16 mmHg in the OD without medication and 25 mmHg in the OS with three medications, respectively. The AC angle was wide open in both eyes (OU), and pseudoexfoliation (PE) was observed at the pupillary margin in the OS. An Emery-Little grade 1 nuclear cataract was observed OU. Vertical cup-to-disc ratios were 0.4 OD and 1.0 OS with pale optic nerve head color OS. The visual field mean deviation was +0.90 decibels (dB) OD and -7.66 dB OS, and the foveal sensitivity was 38 dB OD and 25 dB OS with a Humphrey visual field analyzer (Carl Zeiss Meditec, Dublin, CA; central 30-2 program). Optical coherence tomography (OCT) (RS3000 Advance 2, Nidek, Gamagori, Japan) detected thinning of retinal nerve fibers OS. 

For IOP reduction, filtration surgery with PFM implantation was performed for exfoliation of glaucoma OS (Video 1).

**Video 1:** Surgical findings of the initial PFM implantation PFM: PreserFlo MicroShunt

A nine to 12 o'clock limbal conjunctival incision was made in the OS, and 0.04% mitomycin C was applied subconjunctivally with a sponge for three minutes, followed by rinsing with a balanced salt solution. A specialized double-step knife included in the PFM package was used to create a scleral tunnel to the AC from a distance of 3 mm from the limbus. The PFM was inserted into the scleral tunnel, and the aqueous humor outflow from the PFM was checked with a surgical sponge. The Tenon tissue and conjunctiva were then reapproximated to the limbus with 10-0 Vicryl sutures (Johnson & Johnson, New Brunswick, NJ), and the surgery was completed. Postoperatively, 1.5% levofloxacin (Nipro, Osaka, Japan) and 0.1% betamethasone (Sanbetason, Santen Pharmaceutical Co., Ltd., Osaka, Japan) were applied topically four times daily.

**Figure 1:**
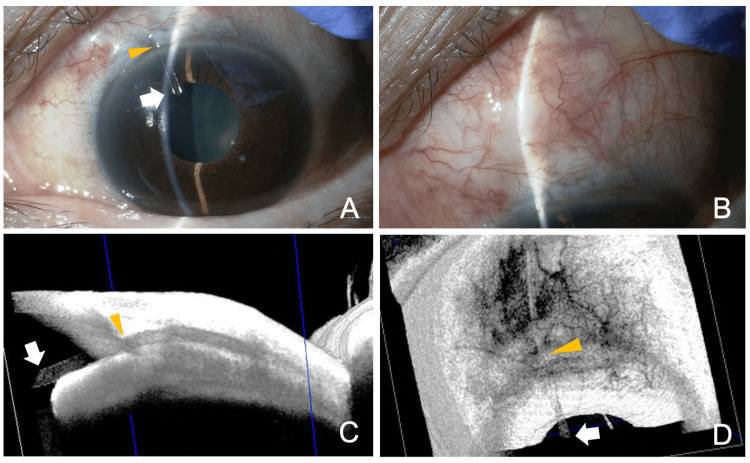
Slit lamp (A, B) and AS-OCT (C, D) findings just before the postsurgical needling procedure, 21 days after the initial PFM implantation. (A) The tip of the PFM is seen in the anterior chamber OS (arrow). (B) The bleb height is relatively low, suggesting restricted filtration. (C, D) AS-OCT clearly shows the position of the device in the anterior chamber OS (arrow). The yellow arrowhead indicates the corneal limbus AS: anterior segment; OCT: optical coherence tomography; PFM: PreserFlo MicroShunt; OS: left eye

On postoperative day five, the BCVA was 0.5 and the IOP was 8 mmHg OS with no anti-glaucoma medication; a well-positioned device in the AC and bleb formation that extended to the fornix were observed. On postoperative day 21, the BCVA was 0.5 and the IOP was 21 mmHg OS with no anti-glaucoma medication, indicating an increased IOP OS. The tip of the PFM could be seen in the AC OS (Figure 1A, arrow), but the filtration bleb had shrunk (Figure 1B).

**Figure 2:**
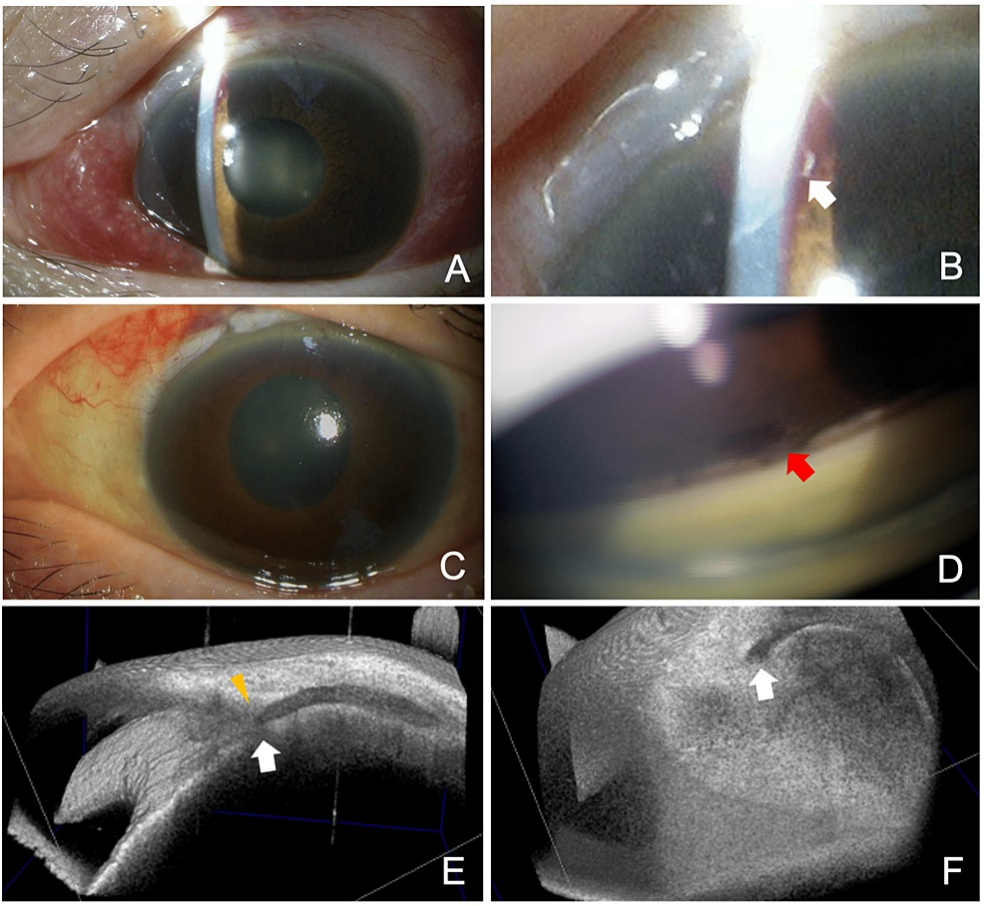
Slit lamp (A–D) and AS-OCT (E, F) findings after the needling procedure (A) Just after the needling procedure, the length of the tube presented in the anterior chamber becomes shorter. (B) A blood clot around the device tip is seen in the OS (arrow). (C) Eight days after the needling procedure, corneal edema due to high IOP is observed. (D) Gonioscopy reveals the absence of the device tip in the anterior chamber and the formation of peripheral anterior synechia where the device tip was placed (red arrow). (E, F) AS-OCT shows the position of the deviated PFM subconjunctivally. The arrow indicates the tip of the PFM, and the yellow arrowhead represents the corneal limbus. AS: anterior segment; OCT: optical coherence tomography; IOP: intraocular pressure; PFM: PreserFlo MicroShunt; OS: left eye

Anterior segment OCT (AS-OCT) (CASIA2 Advance, Tomey Corporation, Nagoya, Japan) also showed that the tip position of the PFM relative to the corneal limbus was appropriate in the AC OS (Figures 1C, 1D). Since the IOP was high and the filtration bleb was shrinking the OS, a needling procedure was performed on the same day. Under the surgical microscope, using a 26-gauge sharp needle connected to a 2.5-ml syringe, after a small amount of 2% lidocaine was injected subconjunctively, the adhesion around the distal end of the PFM was released by moving the needle tip several times. After the procedure, the bleb size increased, and the eyeball became softer. By slit-lamp examination, the device tip moved toward the corneal limbus and became less visible in the AC OS (Figures 2A, 2B).

On postoperative day 29 (i.e., eight days after needling), the patient returned to our hospital because she experienced decreased vision in the OS at the time of waking in the morning. The BCVA was 0.02, and the IOP was 60 mmHg OS. Slit-lamp examination of the OS revealed corneal edema (Figure 2C) with deep AC depth; the tip of the PFM was not observed in the AC. Gonioscopy of the OS also failed to reveal the tip of the PFM in the AC, and a peripheral anterior synechia (PAS) was observed at the site where the PFM had been inserted (Figure 2D, red arrow). An AS-OCT revealed a deviated PFM subconjunctivally (Figures 2E, 2F, and Video 2).

**Video 2:** Dislocated PFM assessed by AS-OCT AS: anterior segment; OCT: optical coherence tomography; PFM: PreserFlo MicroShunt

**Figure 3:**
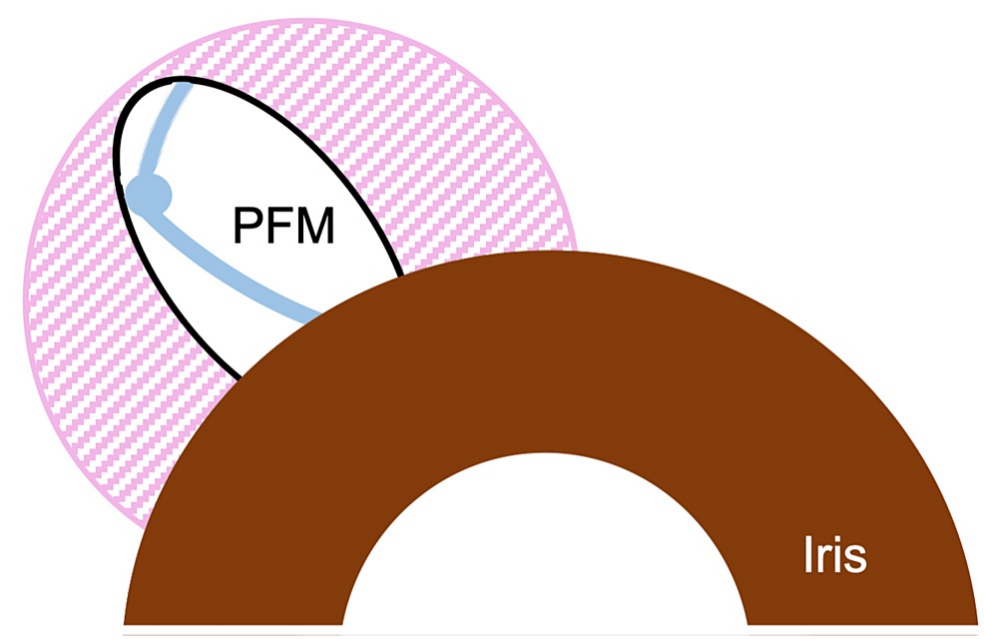
Schematic drawing of the dislocated PFM in the subconjunctival space The PFM can be bent in the space restricted by the corneal limbus/scleral tunnel and the bleb. The pink diagonal lines represent subconjunctival adhesion with the sclera. PFM: PreserFlo MicroShunt Image credits: Kana Murakami and Masaki Tanito.

As seen in the AS-OCT imaging (Figure 2F), the PFM can be bent in the narrow space restricted by the corneal limbus/scleral tunnel and the encapsulated bleb (Figure 3).

**Figure 4:**
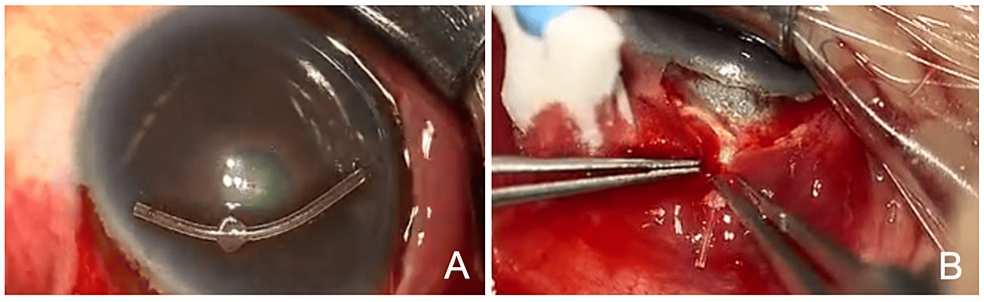
Findings of the reoperation (A) The explanted device is curved. (B) After the removal of the dislocated device, a new PFM is implanted through a newly created scleral tunnel. PFM: PreserFlo MicroShunt

She was diagnosed with increased IOP due to the complete dislocation of the PFM, and surgical revision was planned five days later. Until the revision surgery, twice-a-day timolol and dorzolamide were prescribed. Intraoperative findings showed that the tip of the PFM had not completely exited the scleral tunnel but had moved outward; as observed with AS-OCT, the PFM was found to be deformed (Figure 4A, Video 3).

**Video 3:** Surgical findings of dislocated PFM explanation and new PFM implantation PFM: PreserFlo MicroShunt

**Figure 5:**
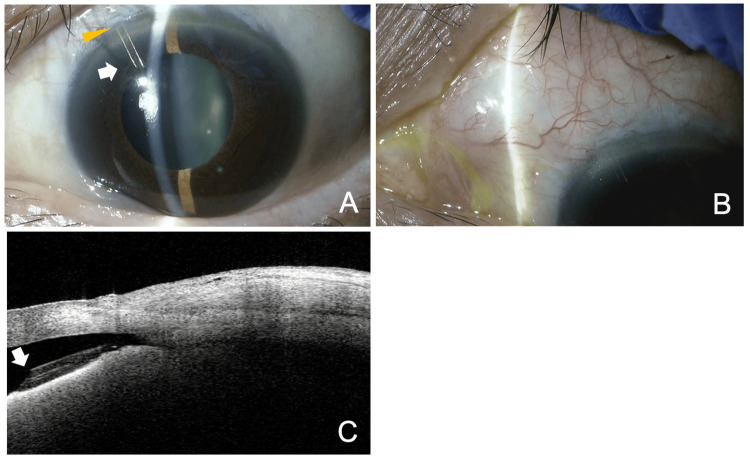
Slit lamp (A, B) and AS-OCT (C) findings three months after the explanation of the dislocated PFM and second PFM implantation (A, C) A well-positioned device is observed. (B, C) A filtering bleb is visible. The arrow indicates the tip of the PFM, and the yellow arrowhead represents the corneal limbus. AS: anterior segment; OCT: optical coherence tomography; PFM: PreserFlo MicroShunt

The deformed PFM was removed, a new scleral tunnel was created next to the original tunnel, and another PFM was inserted (Figure 4B). Postoperatively, 1.5% levofloxacin (Nipro, Osaka, Japan) and 0.1% betamethasone (Sanbetason, Santen Pharmaceutical Co., Ltd.) were applied topically four times daily. On postoperative day one, the IOP was 8 mmHg OS with no anti-glaucoma medication. At the last visit, three months after reoperation, the BCVA was 0.8 and the IOP was 19 mmHg OS with no anti-glaucoma medication. The tip position of the PFM was appropriate (Figure 5A), and a filtration bleb had formed (Figure 5B). The AS-OCT showed the well-positioned tip of the PFM in the AC OS (Figure 5C).

## Discussion

We report a case of PFM dislocation following a postsurgical needling procedure for reduced bleb function. In a meta-analysis study, among various glaucoma filtration surgeries including trabeculectomy, deep sclerectomy, Ex-PRESS Shunt (Alcon Laboratories, Fort Worth, TX), Ahmed Glaucoma Valve (New World Medical, Rancho Cucamonga, CA), Baerveldt Glaucoma Implant (Johnson & Johnson Vision, Santa Ana, CA), Molteno implant (Molteno Ophthalmic Limited, Dunedin, New Zealand), Xen Gel Stent (Allergan INC, Dublin, Ireland), and PFM, the rate of postsurgical needling procedures after all surgeries was 0.33/100 patient-months, while the rate for PFM alone was 0.08/100 patient-months [10]. Although the rate is relatively low, the needling procedure may be required after PFM implantation, as in our case. PreserFlo MicroShunt dislocation is a unique complication associated with postsurgical needling procedures that have not been widely reported in the literature.

**Figure 6:**
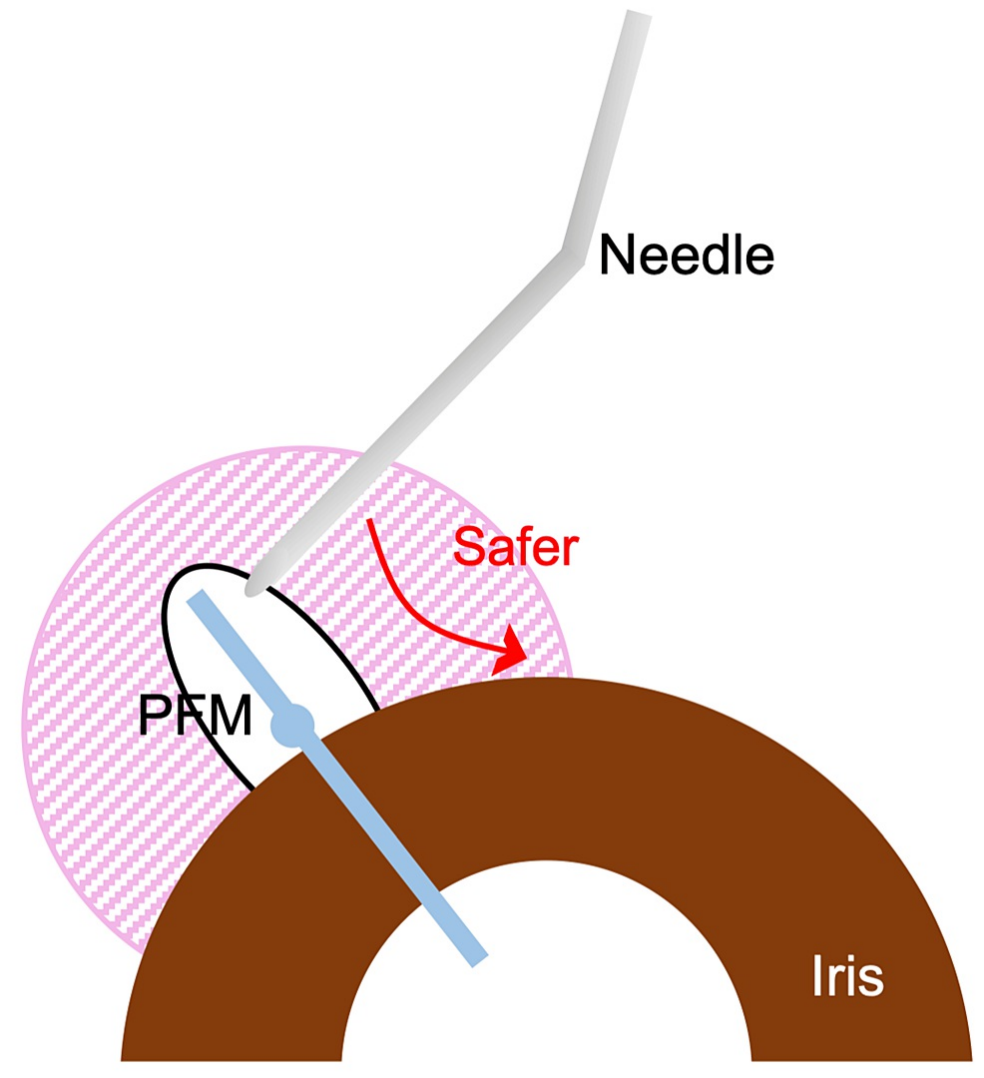
A schematic drawing of the direction of needle tip movement during the needling In eyes implanted with PFM, to avoid device dislocation, the needle tip is moved toward the corneal limbus (red arrow) instead of the fornix, which is safer. PFM: PreserFlo MicroShunt Image credits: Kana Murakami and Masaki Tanito

During the needling procedure, the treating physician inadvertently moved the needle tip from the limbal side to the fornix side, following the usual procedure after trabeculectomy. The fixation of the PFM to the sclera was not firm, which resulted in the dislocation of the PFM. In such cases, an initial option for repositioning the device could involve pushing back the device tip into the AC by manipulating it with a cotton swab or forceps over the bleb. Alternatively, pulling the device tip further into the AC using intracameral forceps inserted through a corneal side port might be a more reliable option, although this would require performing the procedure in an operating room. In our case, since there was a reduction in IOP and the device tip was still visible in the AC, the treating physician decided to observe rather than reposition the device. Unfortunately, this led to a significant increase in IOP when the device completely dislocated from the AC eight days later. The exact reason for further movement of the PFM is unknown, but it may have been related to blinking or rubbing the eye. Once the fin is out of the scleral pocket, the device may move easily. To avoid the complications we experienced, when performing the needling procedure after PFM implantation, treating physicians are recommended to move the needle tip in the opposite direction of the usual trabeculectomy procedure (Figure 6).

Due to the closure of the preexisting scleral tunnel and the deformation of the PFM, a new scleral tunnel was created, and a new PFM was inserted in this case. No further complications were observed, and successful bleb formation was achieved.

## Conclusions

In conclusion, like other glaucoma filtering surgeries, PFM may require postsurgical needling procedures. Needling procedures may cause PFM dislocation and an increase in IOP, resulting in the need for further IOP-reducing procedures. During the needling procedure for eyes implanted with PFM, it seems safer to move the needle tip from the fornix side to the limbal side to prevent PFM dislocation.

## References

[1] Pinchuk L, Riss I, Batlle JF (2016). The use of poly(styrene-block-isobutylene-block-styrene) as a microshunt to treat glaucoma. Regen Biomater.

[2] Baker ND, Barnebey HS, Moster MR (2021). Ab-externo Microshunt versus Trabeculectomy in primary open-angle glaucoma: one-year results from a 2-year randomized, multicenter study. Ophthalmology.

[3] Jamke M, Herber R, Haase MA, Jasper CS, Pillunat LE, Pillunat KR (2023). PRESERFLO ™ MicroShunt versus trabeculectomy: 1-year results on efficacy and safety. Graefes Arch Clin Exp Ophthalmol.

[4] Van Lancker L, Saravanan A, Abu-Bakra M (2023). Clinical outcomes and cost analysis of PreserFlo versus trabeculectomy for glaucoma management in the United Kingdom. Ophthalmol Glaucoma.

[5] Ibarz Barberá M, Martínez-Galdón F, Caballero-Magro E, Rodríguez-Piñero M, Tañá-Rivero P (2022). Efficacy and safety of the PreserFlo MicroShunt with mitomycin C for the treatment of open angle glaucoma. J Glaucoma.

[6] Tanner A, Haddad F, Fajardo-Sanchez J (2023). One-year surgical outcomes of the PreserFlo MicroShunt in glaucoma: a multicentre analysis. Br J Ophthalmol.

[7] Cassottana P, Di Mola I, Ferro Desideri L, Vagge A, Cutolo CA, Traverso CE, Iester M (2022). Blood reflux through a PreserFlo MicroShunt device after needling. J Glaucoma.

[8] Fahy ET, Ho H, Dukht U, Garg A, Lim KS (2022). Conjunctival erosion following a PRESERFLO® MicroShunt procedure. Am J Ophthalmol Case Rep.

[9] Brambati M, Bettin P, Ramoni A, Battista M, Bandello F (2022). A case of endophthalmitis following needling procedure after PRESERFLO(®) MicroShunt implantation. Eur J Ophthalmol.

[10] Marolo P, Reibaldi M, Fallico M (2022). Reintervention rate in glaucoma filtering surgery: a systematic review and meta-analysis. Eur J Ophthalmol.

